# Impact of Sprouted Chickpea Grits and Flour on Dough Rheology and Bread Features

**DOI:** 10.3390/foods13172698

**Published:** 2024-08-26

**Authors:** Andrea Bresciani, Alessio Sergiacomo, Andrea De Stefani, Alessandra Marti

**Affiliations:** Department of Food, Environmental and Nutritional Sciences (DeFENS), Università degli Studi di Milano, Via G. Celoria 2, 20133 Milan, Italy; andrea.bresciani@unimi.it (A.B.);

**Keywords:** pulses, particle size, dough rheology, bread-making

## Abstract

This study investigated the effects of incorporating sprouted chickpeas, at a 25% enrichment level, into bread production as either grits (90% of particles ≥500 µm) or flour (90% of particles ≤250 µm). The focus was to investigate the role of particle size on dough and bread. In addition to the functional, mixing and pasting properties of ingredients, gluten aggregation, mixing, extensional, leavening, and pasting properties of the blends were assessed during bread-making, as well as bread volume and texture. Chickpea particle size influenced water absorption capacity (1.8 for grits vs. 0.75 g/g for flour) and viscosity (245 for grits vs. 88 BU for flour), with flour showing a greater decrease in both properties. With regard to dough properties, dough development time (16.6 vs. 5.3 min), stability (14.6 vs. 4.6 min), and resistance to extension (319 vs. 235 BU) was higher, whereas extensibility was lower (105 vs. 152 mm) with grits, compared to flour. During bread-making, grits resulted in a higher specific volume (2.5 vs. 2.1 mL/g) and softer crumb (6.2 vs. 17.4 N) at all the considered storage times. In conclusion, sprouted chickpea grits can be effectively used as a new ingredient in bread-making favouring the consumption of chickpea, without compromising product quality.

## 1. Introduction

Chickpea (*Cicer arietinum* L.) is a pulse crop globally recognised for its nutritional significance and versatility in culinary applications [1]. With the rising demand for plant-based protein sources and the growing interest in sustainable food production, exploring approaches to enhance the use of chickpeas in food products has garnered considerable attention in recent years [2]. Among various processing techniques, sprouting (also known as germination) has emerged as a promising method to improve the sensory properties, nutritional quality, and functionality of pulses, including chickpeas. During sprouting, the biochemical composition of chickpeas undergoes significant modifications: enzymatic activity increases, leading to the breakdown of complex macromolecules such as proteins, carbohydrates, and lipids into simpler forms, thereby enhancing their digestibility [3]. Moreover, the germination process activates dormant enzymes, resulting in the synthesis of essential nutrients, including vitamins, minerals, and phytochemicals, while simultaneously reducing the levels of anti-nutritional factors such as phytic acid and enzyme inhibitors [4]. Such changes contribute to improved protein quality, increased fibre content, elevated levels of vitamins (e.g., vitamin C and B-complex vitamins), minerals (e.g., iron and calcium), and enhanced antioxidant activity in sprouted chickpeas. Furthermore, sprouted chickpeas exhibit favourable functional properties, including improved water absorption capacity, emulsifying properties, and enhanced sensory attributes, making them a desirable ingredient for various food formulations [5]. Indeed, sprouting also affects the overall structural organisation of proteins (i.e., aggregate formation, thiol/disulfide balance, and the release of peptides), which has a positive effect on the dough mixing and leavening properties of chickpea dough (wheat–chickpea ratio = 80:20), with respect to dough with unsprouted chickpeas [6]. Molecular changes that occur upon sprouting for 48 h have resulted in alterations in the hydration properties of chickpea flour, specifically increased water absorption and water hydration capacities [7]. Changes in functional properties have been attributed to the increased protein solubility upon sprouting, which also improved other physicochemical properties such as the oil absorption capacity and emulsifying activity [8]. However, a longer sprouting time (i.e., 96 h) promoted further changes in chickpea molecular organisation that weakened the gluten network, when 15% sprouted chickpea-enriched dough was subjected to simultaneous mechanical mixing action and an increase in the temperature [9].

Despite the growing interest in sprouted chickpeas from a nutritional and technological point of view, there remains a need to understand the impact of using it in food products and how to manage the factors that impact product quality.

Despite it being well-recognised that particle size should be considered when formulating pulse-based breads, to the best of our knowledge, no information is available on the effect of using chickpea grits instead of flour in bread-making. Understanding how different particle sizes affect the physicochemical and rheological attributes of bread is essential for optimising the formulation and production of baked goods with improved nutritional profiles. Indeed, fine milling may expose a larger surface area, facilitating mixing and integration, while coarse milling could retain larger particles, potentially preserving certain bioactive compounds and fibre content [10]. For this reason, this study aims to investigate the effects of particle size variation (flour versus grits) in sprouted chickpea on dough rheology and bread-making performance. Understanding the role of chickpea flour particle size is a crucial factor in enhancing the rheological properties of dough and the quality of bread, making it a valuable consideration for both researchers and bread manufacturers aiming to produce chickpea-enriched products with good bread-making performance.

## 2. Materials and Methods

### 2.1. Materials

Common wheat flour (CWF; 070 type; 13.5% protein, 0.65% ash, and 14% moisture), sprouted chickpea flour (CPF), and sprouted chickpea grits (CPG) were kindly provided by Molino Quaglia spa (Vighizzolo D’Este, Padova, Italy). The CPF and CPG originated from the same batch of sprouted chickpea (43.8% starch; 20.4% protein; 2.8% ash; 7% moisture) and differed only in terms of their particle size distribution, which was determined by using a mechanical bench sieve (Endecotts Octagon, London, United Kingdom) and is reported in Supplementary Figure S1. Both CPF and CPG were blended with CWF in a 25:75 ratio. 

### 2.2. Methods

#### 2.2.1. Functional Properties of Chickpea Flour and Grits

The oil absorption capacity (OAC), water binding capacity (WBC), and water absorption capacity (WAC) at 25 °C and 90 °C, as well as water solubility index (WSI) at 25 °C and 90 °C, were measured in triplicate using the method described by Bresciani et al. [11]. Specifically, each sample was dispersed in distilled water or sunflower oil (flour–water/oil ratio = 1:10) for WAC and OAC measurements, respectively. The suspension was mixed in a vortex for 30 s and left to rest for 10 min; the procedure was repeated three times. The suspension was then centrifuged (30 min at 2500× *g*): the resulting sediment was incubated at 50 °C for 25 min to calculate the WAC and OAC, which were expressed as g/g, and the supernatant was dried at 105 °C for 12 h to calculate the WSI, which was expressed as g/g. The WAC and WSI were also measured using hot distilled water (90 °C) instead of water at room temperature. Finally, the WBC was assessed by dispersing the sample in distilled water (flour–water ratio = 1:10). After mixing in a vortex for 30 s and resting for 10 min (the operation was repeated three times), the sample was centrifugated twice (45 min at 2500× *g*). After each centrifugation, the supernatant was discarded, and the precipitate was dried (50 °C for 25 min) and weighed. The WBC (g/g) was calculated as the difference between the initial weight and the precipitate weight. 

The foaming capacity and stability were assessed in triplicate, as reported by Yasumatsu et al. [12]. The following equations were used:

$$Foaming\;capacity\left(\%\right)=\frac{V_{f}-V_{i}}{V_{i}}\times 100$$

where $V_{f}$ is the volume of the solution after agitation (volume of the foam) and $V_{i}$ is the initial volume of the solution before agitation.

$$Foaming\;stability\left(\%\right)=\frac{V_{t}}{V_{f}}\times 100$$

where $V_{t}$ is the volume of the foam after 30 min and $V_{f}$ is the initial foam volume right after agitation.

#### 2.2.2. Pasting Properties of Chickpea Flour, Grits, and Wheat–Chickpea Blends

Starch gelatinization and retrogradation properties were evaluated using the ViscoQuick (Brabender GmbH and Co KG, Duisburg, Germany), which measures the viscosity variations in samples mixed with distilled water, which underwent rotative movement (250 rpm) and thermal treatments with a cooling and heating rate of +/− 7.5 °C/min. The applied thermal profile included an initial heating of the suspension (10 g sample in 105 mL distilled water) from 30 to 95 °C, followed by 5 min of holding at 95 °C; finally, the slurry was cooled to 50 °C at a speed of −7.5 °C/min. The test was carried out in duplicate and one representative curve for each sample was shown.

#### 2.2.3. Mixing Properties of Chickpea Flour and Grits, and What-Chickpea Blends

The mixing properties of chickpea flour and grits were measured in triplicate using the Farinograph-E (Brabender GmbH & Co. KG, Duisburg, Germany) equipped with a 50 g mixing bowl at a constant hydration level (i.e., 50% on dry matter), as developed by Bresciani et al. [13]. The mixing properties of blends were performed in triplicate following the ICC (International Association for Cereal Science and Technology) method n. 115/1 [14]. One representative curve for each sample was shown.

#### 2.2.4. Gluten Aggregation Properties of Wheat–Chickpea Blends

The aggregation kinetic properties of wheat gluten proteins when CPF or CPG were added (chickpea–wheat ratio = 25:75) was measured using the GlutoPeak (Brabender GmbH and Co KG, Duisburg, Germany), as reported by Chandi and Seetharamn [15]. During the test, the sample (8.5 g) in the presence of 9.5 g CaCl_2_ 0.5 M was subjected to intense mechanical action (1900 rpm), allowing the formation of the gluten network, and a change in torque was recorded. The test was carried out in triplicate and one representative curve for each sample was shown.

#### 2.2.5. Extensional Properties of Wheat–Chickpea Blends

Both uniaxial and three-dimensional extension properties were measured for dough samples. The former were evaluated using an Extensograph (Brabender GmbH and Co KG, Duisburg, Germany), according to the AACCI (American Association of Cereal Chemists International) method (AACCI 54-10.01) [16]. The latter was assessed using an Alveograph (Chopin Technologies, Villeneuve La Garenne Cedex, France) according to the ICC method n. 121 [15]. For the Extensograph test, two doughs were prepared and, from each of them, two sub-samples were analysed, with one representative curve shown for each sample. For the Alveograph test, the flour was mixed with a 2.5% sodium chloride solution and the amount of water was determined using the following formula: water quantity (mL) = (14 − % moisture content of the flour) × 250. Two doughs were prepared and, from each of them, five sub-samples were analysed; from each, cure tenacity (P), extensibility (L), and strength (W) were considered.

#### 2.2.6. Leavening Properties of Wheat–Chickpea Blends

Dough samples were prepared in the mixing bowl of an Alveograph, adding 250 g of flour, distilled water (as determined in the Farinograph test), olive oil (3.3 g/100 g flour), dry yeast (2.7 g/100 g flour), sugar (2.7 g/100 g flour), and salt (1.5 g/100 g flour). Once the dough was formed, it was loaded into a Rheofermentometer (Chopin Technologies, Villeneuve La Garenne Cedex, France) to measure the dough’s development and gas production and release during leavening for 180 min. The test was carried out in duplicate and one representative curve for each sample was shown.

#### 2.2.7. Bread-Making

Baking was carried out using 300 g of flour and the formula reported in Section 2.2.6. Firstly, water and yeast were mixed for 2 min; after that, flour, sugar, salt, and oil were added and mixed for 15 min of intermittent mixing (3 s of stop every 27 s of mixing) and for 7 min of regular mixing. After that, the dough was leavened for 1 h and 30 min at 30 °C and 80% relative humidity. Six portions of 80 g each were hand formed and transferred into pans (5.7 × 10.3 × 2.5 cm) that were greased with virgin olive oil, leavened for 15 min (at 30 °C and 80% relative humidity), and baked at 160 °C for 40 min. Bread-making was carried out in duplicate and, from each batch, 6 bread loaves were produced. Breads analysed at t1 and t2 were stored in a high-density polyethylene bag at room temperature until analysis.

#### 2.2.8. Bread Characterisation

The colorimetric analysis was performed using the Chroma Meter CR 210 colorimeter (Minolta Camera Co., Osaka, Japan). For each loaf, five determinations for the crust and five for the crumb were carried out, at different points of the surface. The colour was expressed using absolute chromaticity indices in the CIE-L* a* b* space. The bread specific volume was measured as the volume–weight ratio of 12 loaves. The former was determined using the AACC 10-05.01 method [16], whereas an electronic scale (Europe 1700; Gibertini elettronica SRL, Novate, Italy) was used for the latter. Crumb moisture content was detected using a thermobalance (Radwag—Wagi Elektroniczne, Chorzòw, Poland). The test was carried out by weighing 5 g of crumb, homogeneously distributed on an aluminium support. The moisture value was recorded when, at 130 °C, the weight was stable (defined as a change in weight lower than 1 mg every 10 s). Crumb moisture was measured in four loaves at each considered time (t0, t1, and t2). Finally, texture analysis was performed using a texture analyzer (TA-TX Plus, Stable Micro System, Surrey, UK), equipped with a 100 N load cell and a 25 mm diameter cylindrical probe (TA-11), and the speed was set to 1 mm/s. Samples were prepared by slicing the bread in 2.5 cm wide slices, obtaining 2 slices from 4 loaves for a total of 8 measures per type of bread, at any considered time (t0, t1, and t2).

#### 2.2.9. Statistical Analysis

Data are expressed as mean ± standard deviation. A *t*-test was carried out when chickpea grits and chickpea flour were compared. When wheat was also considered, a one-way analysis of variance (ANOVA) was carried out using the Statgraphics Plus 5.1 (Statpoint Inc., Warrenton, VA, USA) software. Samples (doughs or bread) were considered as factors and significant differences between means were detected using the Tukey HSD (Honestely Significant Difference) test at *p* < 0.05.

## 3. Results and Discussion

### 3.1. Chickpea Flour and Grits Characterisation

In the first part of the study, we characterised both grits and flour from sprouted chickpea for their hydration, foaming, pasting, and mixing properties, to obtain information on the effect of chickpea particle size on the interactions with solvents (i.e., water and oil). Since both flour and grits were obtained from the same batch of sprouted chickpeas, the observed and discussed differences were not due to chemical composition but as effect of molecular interactions affected by particle size. Specifically, sprouted chickpea was milled to obtain flour with 90% of the particles measuring less than 250 μm and grits were defined as having 90% of the particles measuring more than 500 μm (Supplementary Figure S1). 

#### 3.1.1. Functional Properties of Chickpea Flour and Grits

The functional properties of grits and flour are shown in Table 1. Functional properties refer to several properties of a food ingredient that might affect its behaviour during food production. In this frame, we assessed interactions with water (both at 25 °C and 90 °C) and oil, as well as the ability to form and stabilised foams; all the mentioned properties play an important role in the production of bread or other leavened products. 

The goal of this activity was to compare the functional properties of chickpea grits and flour, in order to elucidate the potential impact of particle size. The functional properties of the wheat flour used for the blends have been previously reported by Bresciani et al. [11].

Compared to chickpea flour, grits exhibited higher water absorption and binding capacities, and a low water solubility index, suggesting more interaction with water. 

On the other hand, the lower oil absorption capacity in grits, compared to flour, would suggest fewer hydrophobic interactions in grits and differences in the way hydrophobic amino acids may interact with hydrocarbon chains of fats [17]. Our results are in the range of those reported by Kaur and Singh [18], who stated that the water absorption capacity of different chickpea cultivars ranged from 1.33 to 1.47 g/g. With regard to the oil absorption capacity, which is related to mouth feel and helps to retain the flavours, our data were slightly lower than those reported by a previous study (i.e., from 1.05 to 1.17 g/g; [18]).

At 90 °C, the ability to absorb water was similar between the samples, because of complete starch gelatinization, but water solubility index was lower in grits indicating a higher granular integrity/structure stability during heating, likely due to a higher strength of the hydrogen bonding between the granules [19]. Other authors have been related low water solubility index to low damaged starch content [20], suggesting that the lower the particle size, the higher the starch damage and the higher the water solubility index. Comparing our data with those reported in the literature, besides differences in methodology between the studies, it should be considered that our samples were subjected to a sprouting process that likely promoted starch degradation via the amylases developed during sprouting. 

Finally, flour showed a lower foaming capacity than grits (Table 1). Generally, a low foam volume but a high foam stability indicates the production of relatively thick foams [18]. Both samples showed very high and similar foam stabilities (>90%) after 30 min of storage. The good foam stabilities of both samples suggest that proteins that are soluble in the continuous phase (water) are very surface-active in both grits and flour samples.

#### 3.1.2. Pasting Properties of Chickpea Flour and Grits

The goal of this activity was to compare the pasting properties of chickpea grits and flour to elucidate the potential impact of particle size. The pasting properties of the wheat flour used for the blends are reported in Section 3.2.5.

**Figure 1:**
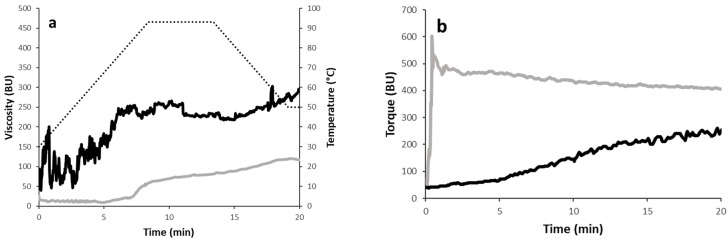
Rheological properties of sprouted chickpea grits (black solid line) and flour (grey solid line): (**a**) pasting properties (dotted line refers to temperature profile); (**b**) mixing properties (at 50% hydration). BU, Brabender units. A representative curve for each sample is reported.

The changes in viscosity during the heating and cooling steps are shown in Figure 1a. As the temperature increased, the viscosity increased too, but the two samples followed different trends. Specifically, the beginning of gelatinization (i.e., the beginning of the increase in viscosity) occurred at lower temperature in the grits compared to the flour (68 °C ± 0.7 versus 77 °C ± 0.4). Moreover, during heating, grits exhibited a higher maximum viscosity than flour (245 ± 25 BU vs. 88 ± 10 BU), suggesting a higher gelatinization tendency, at least in the conditions used during the analysis. The results agree with the high hydration properties of grits shown in Table 1. During cooling, the viscosity further increased due to the reorganisation of gelatinized starch granules. The samples reached different final viscosity values (193 ± 16 BU vs. 125 ± 11 BU for grits and flour, respectively), which indicates the ability of the material to form a viscous paste. However, no differences in setback values were observed (47 ± 8 BU vs. 39 ± 2 BU, for grits and flour, respectively), suggesting a similar retrogradation tendency or syneresis upon the cooling of cooked pastes. Although a few studies have reported the pasting properties of chickpea flours or other pulses considering the effect of particle size [18,21,22], none of them included grits as a raw material. For example, Ahmed et al. [23] evaluated the impact of particle size, in the range of 63–210 μm, of some Indian and Turkish lentil cultivars. The peak viscosity of Indian flour exponentially increased with a decreasing particle size, whereas an opposite trend was reported for the Turkish sample. Such differences were attributed to starch granule sizes that decreased with decreasing flour particles of the Indian varieties but remained constant for the Turkish cultivars. The milling system applied in our study did not involve the removal of any fractions; thus, there is no rationale for a change in starch granule size. Therefore, aspects other than starch granule size should be considered. The hydration properties of grits reported in Table 1 might account for the differences in pasting profiles.

#### 3.1.3. Mixing Properties of Chickpea Flour and Grits

The changes in consistency/torque upon mixing a dough prepared with either sprouted chickpea grits and flour at a constant hydration level (50%) are shown in Figure 1b. Chickpea flour exhibited a rapid increase in torque that can be explained as a rapid water uptake from non-starch polysaccharides and/or damaged starch. After that, dough torque was quite constant, ranging from about 480 to 400 UB. Similar profiles were detected in red lentils [24]. In the case of chickpeas, the dough achieved maximum torque values of around 300 BU, which reset to zero after 12 min of mixing [13].

Chickpea grits showed a different mixing profile: the sample hydration was much slower and seemed to not complete within the 20 min of analysis. Moreover, the consistency of the dough was lower (i.e., about 200 BU), suggesting a different hydration capacity. 

Usually, 500 BU is used as the optimal target for wheat dough. In the case of gluten-free formulations, a lower dough consistency is preferred, to assure good performances during leavening, as ingredients with a high water affinity level are included in the recipe [25].

### 3.2. Dough Rheology

In the second part of the study, grits and flour from sprouted chickpea were added to a common wheat flour (in the ratio of 25:75). A substitution level of 25% represented the maximum limit, to avoid compromising the quality of bread produced with chickpea flour, as previous studies have demonstrated that a substitution level of 30% can significantly deteriorate the properties of the bread [26]. The rheological properties of the blends were assessed to mimic dough behaviour during the main step of bread-making process: the mixing step was assessed using a Farinograph and the GlutoPeak, moulding/shaping using an Extensograph and an Alveograph, leavening using a Rheofermentometer, and baking using the Micro-Visco-Amylograph. The main indices are reported in Supplementary Table S1.

#### 3.2.1. Gluten Aggregation Properties of Wheat–Chickpea Blends

**Figure 2:**
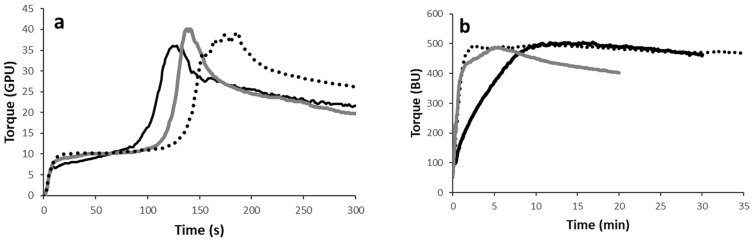
Rheological properties of wheat alone (black dotted line) or in mixture at 25% with sprouted chickpea grits (black solid line) and flour (grey solid line): (**a**) gluten aggregation properties; (**b**) mixing properties (at 50% hydration). BU, Brabender units; GPU, GlutoPeak units. A representative curve for each sample is reported.

The impact of chickpea grits and flour on wheat gluten aggregation properties was assessed using a GlutoPeak test, and the results are shown in Figure 2a. As the gluten is formed, the device registers an increase in torque, until a maximum value that corresponds to the maximum gluten formation. After this point, the torque decreases due to the gluten breakage because of the effect of the high shear stress.

Adding chickpea (either as grits or flour) to wheat modified the aggregation kinetics of gluten proteins, resulting in a faster aggregation (i.e., the peak maximum time—which is the time at which the maximum torque occurs—decreased) and dough weakening (as the peak became sharper). Some differences in gluten aggregation properties occurred based on the chickpea particle size used (grits vs. flour). Indeed, although the presence of grits resulted in a lower maximum peak, this blend seemed to be more stable during mixing. 

#### 3.2.2. Mixing Properties of Wheat–Chickpea Blends

The mixing properties of wheat–chickpea blends are reported in Figure 2b. To achieve a constant consistency of 500 BU, the three samples required different amounts of water. Specifically, the water absorption capacity of wheat dough was about 59% and it increased up to about 62% when 25% chickpea was added, regardless of particle size of chickpea (grits or flour). The increase in water absorption capacity was attributed to increased protein and fibre, which chickpea is rich in. Chickpea grits and flour had an opposite effect on the time needed to achieve the target consistency, which decreased in the case of chickpea flour (indicating dough weakening) and increased when grits were used, suggesting that coarse particle size required more time to fully hydrate proteins for network formation. With regard to the interference of chickpeas with gluten network formation, the results for the dough confirm what was shown by the GlutoPeak test with an excess of water (Figure 2a). Overall dough weakening was observed with the presence of chickpea (as dough stability decreased; Supplementary Table S1), but this was less evident when grits were used instead of flour. 

From a qualitative point of view, using sprouted chickpea flour resulted in a dough that showed stickiness (i.e., it tended to adhere to the internal surface of the bowl) and difficult machinability (i.e., it was difficult both to transfer the dough from the bowl to the shaping system and to give it the desired shape). On the other hand, the dough with chickpea grits was workable without any difficulties. 

Although this is the first time that the use of chickpea as grits has been assessed, previous studies have investigated the impact of chickpea flour (even after sprouting) on mixing properties [27]. As an example, the addition of chickpea flour increased the water absorption capacity and dough development time, while the effect on dough stability depended on the enrichment level: 10% chickpea exhibited higher stability and resistance to mechanical mixing values than the control, whereas these decreased as the substitution level increased from 20% to 30% [26].

#### 3.2.3. Extensional Properties of Wheat–Chickpea Blends

**Figure 3:**
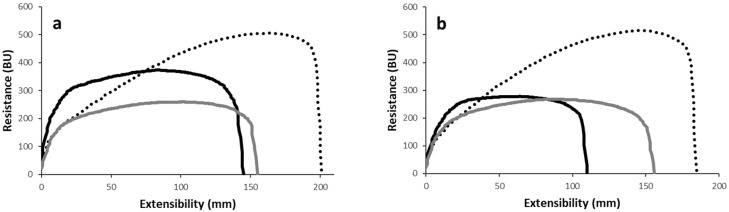
Extensional properties of wheat alone (black dotted line) or in mixture at 25% with sprouted chickpea grits (black solid line) and flour (grey solid line): (**a**) analysis carried out after 45 min of resting; (**b**) analysis carried out after 90 min of resting. BU, Brabender units. A representative curve for each sample is reported.

The extensional properties were assessed, considering the application of both uniaxial and three-dimensional force to the dough. Dough resistance and extensibility during uniaxial extension are shown in Figure 3. The test was carried out at different resting times to evaluate the effect of enzymatic activity on dough properties. After 45 min of resting, the maximum resistance to extension followed the order wheat >> chickpea grits > chickpea flour (Figure 3a), indicating that dough weakness was higher in chickpea flour-enriched dough than in the chickpea grits-enriched sample. When dough resistance was measured at 5 cm of extension, simulating a small deformation, it was seen that adding sprouted chickpea grits to wheat did not alter dough properties; on the contrary, chickpea flour did. These differences were cancelled when a longer resting time (i.e., 90 min) was applied (Figure 3b). Regarding dough extensibility, the order was wheat >> chickpea flour > chickpea grits at any of the considered resting times. Finally, extension energy, which takes into consideration both the resistance to extension and the extensibility, decreased in presence of chickpea, but without differences between grits and flour (141.3 ± 6.8, 71.8 ± 7.6, and 65.4 ± 3.6, for wheat flour, chickpea grits-enriched, and chickpea flour-enriched doughs, respectively; Supplementary Table S1). A decrease in extensibility and resistance as effect of chickpea addition partially confirmed previous findings [27,28,29,30]. The differences between the studies can be related to various factors, including the chickpea enrichment level, the type of wheat flour used to prepare the blends, and the eventual treatments applied to the chickpeas. 

Although a similar amount of water was required to achieve similar consistencies for the two chickpea-enriched doughs (Figure 2b), the dough handling of each sample was quite different (i.e., the ability to form a cohesive non-sticky mass), suggesting a different water distribution between wheat and chickpea components. This is supported by the results shown in Figure 1b: in a dough system (50% hydration), sprouted chickpea flour was able to achieve a higher consistency than grits, likely suggesting more competition with the wheat gluten proteins for water, thus affecting gluten formation. However, this aspect needs further investigation. 

The second approach used to measure dough extensibility was the Alveograph test, which allowed the evaluation of the extensibility and resistance of dough to a three-dimensional extension by air insufflation. The wheat dough’s resistance to air pressure (about 63.9 ± 3.2 mmH_2_0), which is indicative of dough tenacity, increased and decreased when chickpea grits (82.1 ± 5.8 mmH_2_O) and flour (58.8 ± 3.2 mmH_2_O), respectively, were added, suggesting dough rigidity and weakening, respectively. Adding chickpea decreased the dough’s extensibility, with the highest change reported for grits (133 ± 16 mm for wheat dough, 65.6 ± 6.9 mm for chickpea flour, and 30.5 ± 5.5 mm for chickpea grits), confirming the uniaxial extensional data (Figure 3). Finally, the dough strength decreased, regardless of the form of chickpea used (293 ± 18.5 × 10^−4^ J for wheat; 122 ± 7 for flour, and 107 ± 15.6 for grits). In addition to the enrichment level and the particle size (grits vs. flour), the germination time might also have affected the dough’s rheological properties; when 4-day-old sprouted chickpea flour was added to wheat, a decrease in extensibility and strength, but no changes in tenacity, were observed [31].

#### 3.2.4. Leavening Properties of Wheat–Chickpea Blends

**Figure 4:**
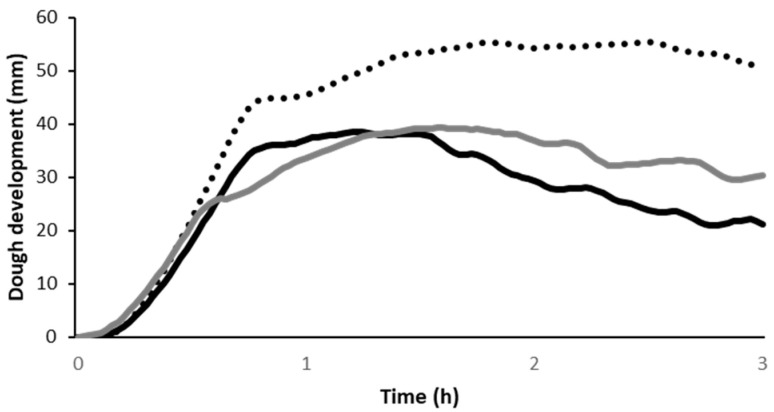
Dough development during the leavening of wheat alone (black dotted line) or in mixture at 25% with sprouted chickpea grits (black solid line) and flour (grey solid line). A representative curve for each sample is reported.

The dough development time and the gas production and retention capacity of the dough samples are reported in Figure 4. Adding sprouted chickpea caused a decrease in dough development and in the time needed to reach the maximum height. The faster dough development might be due to the high sugar content in chickpeas, especially after sprouting [6]. The higher sugar content might also explain the higher gas production of the chickpea-enriched dough. Also, the gas retention capacity was higher in chickpea-enriched doughs, likely due to their reduced extensibility (as shown in Figure 3). Our results confirm those of previous studies on the impact of sprouted chickpea on dough behaviour during leavening [6,31]. However, no differences in dough leavening properties were observed based on the sprouted chickpea particle size.

#### 3.2.5. Pasting Properties of Wheat–Chickpea Blends

**Figure 5:**
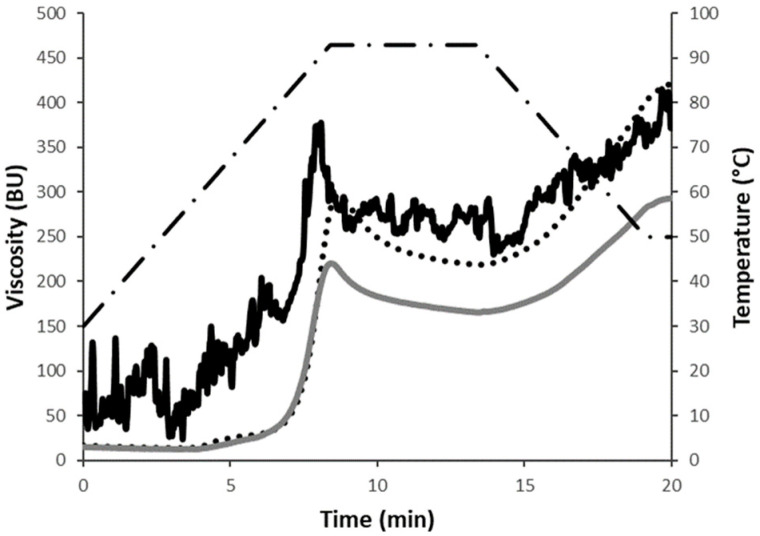
Pasting properties of wheat alone (black dotted line) or in mixture at 25% with sprouted chickpea grits (black solid line) and flour (grey solid line). BU, Brabender units. A representative curve for each sample is reported. Dash-dotted line refers to temperature profile.

Finally, the pasting properties of the blends were assessed to provide information on starch gelatinization and retrogradation affecting bread baking and storage, respectively. Wheat flour showed higher viscosities than the mixture with 25% chickpea flour (Figure 5); the lower starch and higher amylose contents in chickpeas are responsible for decreasing gelatinization and retrogradation properties [32]. In addition, in the present study, chickpeas underwent a germination process that further reduced the properties of starch to gelatinize and retrograde [33]. Regarding the mixture enriched with 25% of chickpea grits, a higher viscosity of the system was seen, compared to the sample with 25% chickpea flour and the sample of wheat flour alone (Figure 5). The higher viscosity in the sample enriched with 25% of chickpea grits was due to the bigger particle size of the grits and to their higher water absorption capacity (Table 1). Investigating the pasting properties of the raw materials has been shown to be a useful tool to gather information on product quality. As an example, the peak viscosity and breakdown of wheat flours is positively correlated with most parameters used in the evaluation of bread quality, and pasting temperature is negatively correlated with bread crumb structure [31].

#### 3.2.6. Bread Characterisation

The addition of chickpeas led to a darkening of the bread crust and crumb (Table 2); this could be due to the presence of a high amount of protein and sugars in chickpea samples and has been seen in other studies [34]. In addition, the chickpeas used in this study underwent a germination process that produced simple sugars, which could have contributed to further browning due to the formation of Maillard compounds [35]. Regarding the crumb, the addition of chickpeas led also to yellowing, confirming previous studies’ results [36]. However, crumb yellowness was more intense in the case of the flour-enriched bread than in the case of the bread enriched with grits. The increase in degree of lightness and the decrease in redness (a) and yellowness (b) with the decrease in particle size was measured for the chickpea flour and the change in colour was found to be related to the increase in the surface area of the chickpea powder after the reduction in particle size and loss of pigments during milling [23,37].

As expected, adding chickpeas to wheat caused a significant reduction in specific volume, due to both gluten dilution and gluten interruption. However, it was possible to observe an effect of particle size, since smaller particle size (i.e., flour) led to a higher reduction in specific volume than coarse particles (i.e., grits). The addition of sprouted chickpeas also decreased the crumb softness (Table 2); however, using chickpea grits instead of chickpea flour has a lesser worsening effect on the textural features of bread, in agreement with the data on specific volume. The differences in crumb texture are not related to the differences in moisture (Table 2). The better behaviour of grits-enriched bread could be due to the larger particle size of the grits that therefore ensure the formation of a gluten network that envelops the large inclusions. On the other hand, the small particles of the flour are homogeneously organised along the protein network, decreasing its properties and worsening its performance during bread-making [38]. 

When using chickpea at a 15% enrichment level, the sprouting process did not affect bread quality [8]. Among other aspects, particle size is a critical parameter in flour milling processes, impacting the quality of the final product [39,40]. Finer-milled pulse flours tend to have greater starch damage, a lower water absorption capacity, and higher peak and final viscosities, resulting in bread with better scores and a tighter, less open crumb structure. However, particle size did not affect bread volume or sensory properties when a 20% enrichment level was used [40]. These differences between the studies might be due to differences in the chemical and/or physical compositions of the raw materials, as well as different particle size ranges considered. In this study, the better baking performance of grits instead of flour was in line with the rheological properties of the related dough. Overall, the results suggested that chickpea grits weakened the gluten network, but only in specific areas of the dough, whereas chickpea flour was homogenously incorporated into the wheat matrix, causing disruptions in the continuous phase of the wheat matrix.

**Figure 6:**
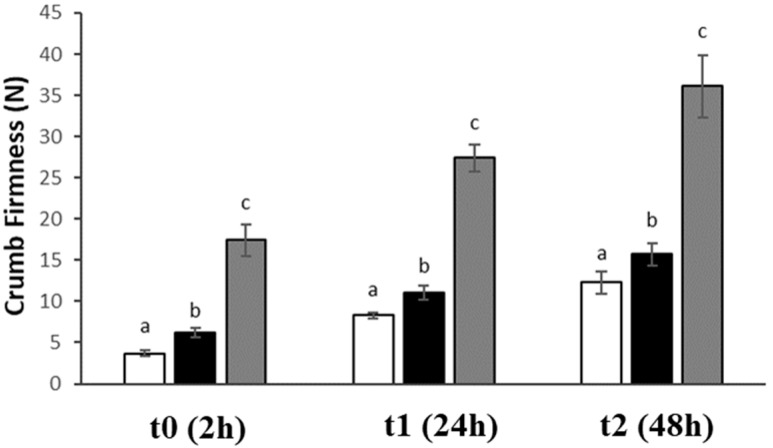
Crumb firmness of bread prepared with wheat (white column), 25% chickpea grits (black column), and 25% chickpea flour (grey column). Analyses were carried out at t0 (2 h), t1 (24 h), and t2 (48 h). Data are expressed as mean ± standard deviation (n = 8). Different letters at the same storage time indicate significant differences (one-way ANOVA, Tukey’s HSD test, *p* ≤ 0.05).

Finally, adding chickpeas (both as grits and flour) led to an improvement in product shelf life. Specifically, the firmness of wheat bread increased by approximately 2.3 times after two days, while the bread containing chickpea grits and flour saw an increase of about 1.5 times and 1 time, respectively, after two days (Figure 6). Starch retrogradation is one of the main factors affecting the loss of crumb softness during storage [41]. However, for this set of samples, other aspects rather than starch retrogradation should be considered, since no relation between setback values (Supplementary Table S1) and crumb firmness (Figure 6) was shown.

The differences between wheat and chickpea-enriched breads could be due to the presence of lipids in chickpeas that limit starch retrogradation, thus slowing down the bread staling [42]. In addition, it has been shown that using sprouted grains in bread formulation decreases bread staling [43].

## 4. Conclusions

Taking into consideration the need to increase the consumption of pulses, due to their nutritional composition and health benefits, this study elucidated the role of chickpea particle size, specifically grits (90% of particles ≥500 µm) or flour (90% of particles ≤250 µm), in bread-making performance. The enrichment of bread using 25% of sprouted chickpeas caused an expected worsening of the dough properties and bread performance, but chickpea grits seemed to perform better than chickpea flour. Specifically, compared to flour, the use of grits increased the dough development time and dough stability during mixing, likely by creating a more tenacious (and stiff) dough. The formulation using sprouted chickpea grits achieved a higher specific volume and had a crumb firmness more similar to wheat bread, compared to the bread made with sprouted chickpea flour. Future studies will assess consumers’ acceptance of the reformulated breads.

Overall, our findings suggest that sprouted chickpea grits hold significant potential as a valuable ingredient in bread production, contributing to better food options in terms of composition (e.g., protein and fibre levels) and promoting sustainable agricultural practices by utilising chickpeas, a legume known for its environmental benefits.

## Figures and Tables

**Table 1:** Functional properties of sprouted chickpea grits and flour.

		Grits	Flour
Absorption properties (25 °C)	Water absorption capacity (g/g)	1.8 ± 0.1	0.75 ± 0.01 ***
	Oil absorption capacity (g/g)	0.69 ± 0.01	0.91 ± 0.02 ***
	Water solubility index (g/g × 100)	1.37 ± 0.08	2.06 ± 0.05 ***
	Water binding capacity (g/g)	1.62 ± 0.01	0.69 ± 0.01 ***
Absorption properties (90 °C)	Water absorption capacity (g/g)	2.26 ± 0.13	1.96 ± 0.17 ^n.s.^
	Water solubility index (g/100 mL)	1.39 ± 0.02	1.98 ± 0.13 **
Foaming properties	Foaming capacity (%)	39.3 ± 1.2	32.7 ± 1.2 **
	Foaming stability (%)	95.6 ± 1.8	93 ± 2 ^n.s.^

Data are expressed as mean (n = 3) ± standard deviation. Asterisks in the same row indicates significant differences between the samples (*t*-test; **, *p* < 0.01; ***, *p* < 0.001); ^n.s.^, not significant.

**Table 2:** Bread-making performance of wheat and wheat-chickpea blends.

		Wheat	25% Chickpea Grits	25% Chickpea Flour
		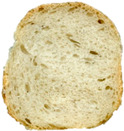	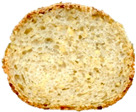	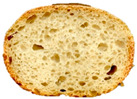
Crust colour	L*	57.4 ± 4.6 a	50.1 ± 5.1 b	49.1 ± 3.3 b
	a*	13.9 ± 2.1 a	16.2 ± 1.7 b	18.5 ± 1.7 c
	b*	30.7 ± 1.7 a	28 ± 3 b	30.9 ± 2.3 a
Crumb colour	L*	68.6 ± 1.7 a	65.04 ± 1.44 b	58.9 ± 1.6 c
	a*	1.97 ± 0.15 a	2.97 ± 0.29 b	3.7 ± 0.3 c
	b*	10.7 ± 0.7 a	15.4 ± 1.2 b	16.6 ± 0.8 c
Specific volume (mL/g)		3.1 ± 0.1 a	2.5 ± 0.1 b	2.1 ± 0.1 c
Crumb moisture (%)	t0	42.4 ± 0.1 a	43.1 ± 0.1 b	43.3 ± 0.1 b
	t1	41.4 ± 0.4 a	42.3 ± 0.2 b	42.2 ± 0.2 b
	t2	40.2 ± 0.2 a	40.5 ± 0.2 ab	40.8 ± 0.3 b

Data are expressed as mean ± standard deviation. Different letters in the same row correspond to significant differences (one-way ANOVA, Tukey’s HSD test, *p* ≤ 0.05). L*, brightness; a*, degree of red–green, b*, degree of yellow–blue; t0, 2 h; t1, 24 h; t2, 48 h. A representative loaf for each sample is reported.

## Data Availability

The original contributions presented in the study are included in the article/Supplementary Material, further inquiries can be directed to the corresponding author.

## Supplementary Materials

The following supporting information can be downloaded at: https://www.mdpi.com/article/10.3390/foods13172698/s1, Figure S1: Particle size distribution of sprouted chickpea grits (black column) and flour (grey column); Table S1: Rheological properties of wheat alone or in mixture at 25% with sprouted chickpea grits and flour.
