# Supplementary material for: Impact of Sprouted Chickpea Grits and Flour on Dough Rheology and Bread Features
Source: Foods. 2024 Aug 26;13(17):2698. doi: 10.3390/foods13172698 (PMC11394579; doi:10.3390/foods13172698)
Supplement: Supplementary file 1 [file foods-13-02698-s001.zip › foods-3165975-supplementary.pdf]

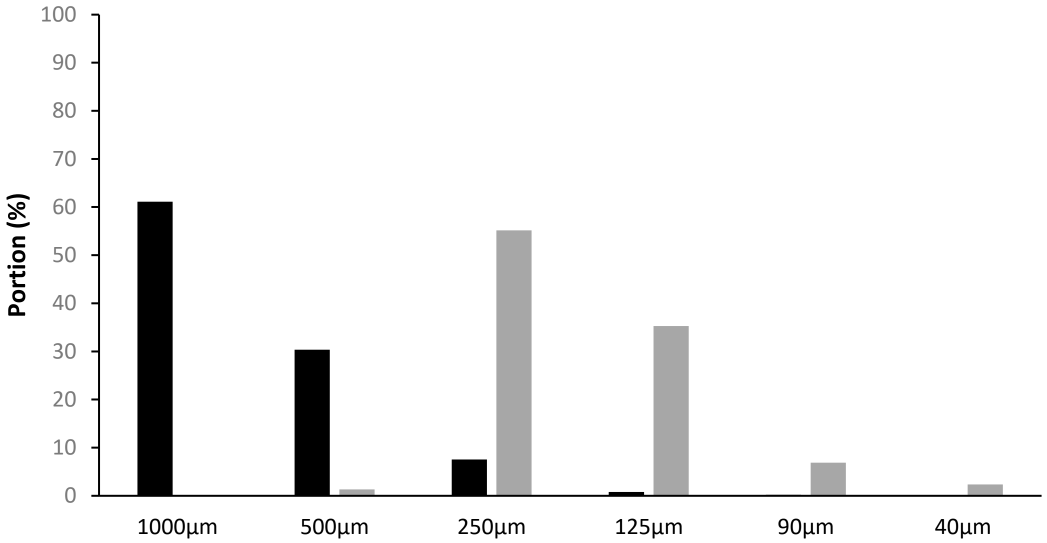

Supplementary Figure S1. Particle size distribution of sprouted chickpea grits (black column) and flour (grey column).

Supplementary Table S1. Rheological properties of wheat alone or in mixture at 25% with sprouted chickpea grits and flour

			Wheat	25% chickpea grits	25% chickpea flour
**Gluten aggregation properties**		**peak maximum time (s)**	181 ± 6a	129 ± 2b	137 ± 1b
		**torque maximum (GPU)**	40 ± 1a	34 ± 2b	40 ± 0a
		**aggregation energy (cm^2)**	1148 ± 26a	977 ± 74b	1071 ± 24b
**Mixing properties**		**water absorption (%)**	58.9 ± 0.1b	61.8 ± 0.1a	61.7 ± 0.1a
		**dough development time (min)**	11.8 ± 1.2b	16.6 ± 0.4a	5.3 ± 0.2c
		**dough stability (min)**	25.3 ± 0.9a	14.6 ± 0.3b	4.6 ± 0.3c
**Extensional properties**	**45 min uniaxial extension**	**extensibility (mm)**	204.3 ± 4.9a	138.4 ± 8.1c	164 ± 10b
		**resistance (BU)**	302.7 ± 6.4a	319.2 ± 23.5a	234.8 ± 6.9b
		**maximum resistance (BU)**	507 ± 1.4a	344 ± 22.9b	259.3 ± 5.7c
		**energy (cm^2)**	141.3 ± 6.8a	71.8 ± 7.6b	65.4 ± 3.6b
	**90 min uniaxial extension**	**extensibility (mm)**	188.7 ± 4.6a	105 ± 16c	152 ± 4b
		**resistance (BU)**	342 ± 19a	254.8 ± 14.3b	241.6 ± 19.4b
		**maximum resistance (BU)**	548 ± 46.7a	256.5 ± 16.5b	254.3 ± 19.7b
		**energy (cm^2)**	139 ± 11a	41.6 ± 8.4c	60 ± 5b
	**triaxial extension**	**strength (W; *10E-4 J)**	292.7 ± 18.5a	106.5 ± 15.6b	122 ± 7b
		**tenacity (P; mmH20)**	63.9 ± 3.2b	82.1 ± 5.8a	58.8 ± 3.2b
		**extensibility (L; mm)**	133 ± 16a	30.5 ± 5.5c	65.6 ± 6.9b
		**P/L**	0.48 ± 0.07c	2.78 ± 0.56a	0.91± 0.14b
**Pasting properties**		**beginning of gelatinisation (°C)**	65.1 ± 0.1b	67.9 ± 1.8ab	70.05 ± 0.21a
		**maximum hot viscosity (BU)**	292.2 ± 6.6b	377.1 ± 4.3a	224.4 ± 5.4c
		**breakdown (BU)**	76.2 ± 2.5b	127.6 ± 20.6a	55.8 ± 1.4c
		**final viscosity (BU)**	181.2 ± 1.9a	133.7 ± 14.5b	118 ± 1c
		**setback (BU)**	417.6 ± 6.4a	389.1 ± 15.7b	297.8 ± 5.7c
**Leavening properties**		**Porosity time (min)**	52.5 ± 6.4a	-	41.2 ± 7.4a
		**Total gas production (ml)**	1915 ± 196b	2452 ± 12a	2343 ± 3.5ab
		**Gas retention capacity (%)**	81.7 ± 1.8b	99.3 ± 0.1a	96.1 ± 0.9a

Data are expressed as mean ± standard deviation. Different letters in the same row correspond to significant differences (one-way ANOVA, Tukey's HSD test, $p \leq 0.05$). GPU, GlutoPeak Units; BU, Brabender Units.
